# The oral health of heroin drug users: case study in Bosnia and Herzegovina

**DOI:** 10.1186/1471-2458-13-1202

**Published:** 2013-12-19

**Authors:** Zorica Terzic Supic, Ranko Petrovic, Milena Santric Milicevic, Goran Trajkovic, Zoran Bukumiric

**Affiliations:** 1 Institute of Social Medicine, Belgrade University Faculty of Medicine, Belgrade 11 000, Serbia; 2 Health Centre Clinicanova, 11 000 Belgrade, Serbia; 3 Institute for Medical Statistics and Informatics, Belgrade University Faculty of Medicine, 11 000 Belgrade, Serbia

**Keywords:** Oral health, Dental disease, Heroin drug users, Prevention

## Abstract

**Background:**

Injection drug use is a major public health problem. Oral health problems and the appearance of dental disease among injection drug users (IDUs) are caused by their lifestyle. The aim of the present study was to examine the relations between socioeconomic factors, drug use, and oral hygiene habits on the oral health of heroin drug users.

**Methods:**

A cross-sectional survey on oral health was carried out as part of UNICEF’s research on the biological and behaviours survey among injection drug users in Sarajevo, Banja Luka and Zenica in Bosnia and Herzegovina. A sample of 519 IDUs participated in the survey. Respondent Driven Sampling (RDS) was used to obtain the sample. The data were obtained through face–to–face interviews using a structured questionnaire related to socio-demographic characteristics, duration of drug injection, frequency of drug injection in the last month and oral health.

**Results:**

Older participants (OR = 1.06; 95% CI = 1.02 -1.10), part–time employment (OR = 3.57; 95% CI = 1.02 - 12.20) and unemployment (OR = 3.23; 95% CI = 1.23 - 8.33) in comparison to full-time employment as the referent category, and longer duration of drug injection (OR = 1.06; 95% CI = 1.003 - 1.12) were predictors of bad oral health. A higher level of education (OR = 0.56; 95% CI = 0.39 - 0.79), more frequent tooth brushing (OR = 0.59; 95% CI = 0.49 - 0.71), and regular dental checkups (OR = 3.30; 95% CI = 1.42 - 7.67) were predictors of good oral health.

**Conclusions:**

Socioeconomic characteristics of IDUs as well as their lifestyles may contribute to oral health problems. Heroin drug users have specific dental needs, and programmes to improve their oral health should be an integral part of strategies to prevent addictions including treatments and harm reduction programmes.

## Background

Injection drug use (IDU) is a major public health problem, and has serious consequences on the health of drug users including HIV/AIDS, hepatitis B, hepatitis C, tuberculosis, accidental overdose, and anesthesia complications [1-3]. In addition, negative effects are manifested in health care delivery such as unavailability of health services or avoidance and resist by health care providers, and in the community in terms of social exclusion [4].

There are only a small number of published papers on the oral health of injection drug users (IDUs), although dental and oral hygiene problems are common in this population [5-7]. Oral health problems and the appearance of dental disease among IDUs are caused by their lifestyle. Lifestyle factors which have been associated with oral health problems are alcohol and tobacco use, poor nutrition, irregular eating habits, and poor personal hygiene [3,7,8]. Tobacco smoking and drinking causes both separate and interactive periodontal disease, pre-cancerous conditions and oral cancer [6]. Alcohol and heroin (and other opioids) are xerostomic and reduce saliva production which may lead to the development of caries and periodontitis [5-8]. Opiates suppress pain which increases pain tolerance among IDUs and consequently they visit the dentist less frequently [9,10].

Bosnia and Herzegovina is on the central route of Balkan drug trafficking, which is known as the “drug road”. It is used by organized criminal groups to transfer a variety of illegal drugs, including heroin, into and out of Europe [11]. Bosnia and Herzegovina should be viewed in the context of the post-war period, with socioeconomic and demographic changes, growing poverty, and increased unemployment. This situation creates conditions where there is a higher risk of developing problems with drug use and possible health consequences (e.g. HIV/AIDS and other blood-borne and sexually transmitted infections).

Several surveys on drug use were carried out in Bosnia and Herzegovina up to 2007, however, these data were not comparable with European research data and their methodology standards [11]. During this period and particularly later, studies were conducted in children [12] and populations whose behaviour exposed them to a higher risk of HIV infection [13,14]. Bosnia and Herzegovina has an inappropriate legal framework, and regulations as well as weak law enforcement. There is a lack of treatment centers for drug addiction, an insufficient number of professionals, and a lack of technology to fight against drug use [11]. Considering this situation, research into the oral health of IDUs has not been conducted in Bosnia and Herzegovina, and in many other countries such research is rare [5,6].

It is known that the oral health of IDUs is complicated by other factors, including socioeconomic factors, as IDUs spend more and more time and energy obtaining and using the drug. Bad oral health may be related to lifestyle habits among IDUs as they are a socially deprived group and often invisible with regard to health and social services [3]. The aim of this study was to examine the relations between socioeconomic factors, drug use, and oral hygiene habits with regard to the oral health status of heroin drug users.

## Methods

### Design and selection of the IDU sample

A cross-sectional survey was carried out as part of UNICEF’s research on the biological and behaviours survey among injection drug users in Sarajevo, Banja Luka and Zenica in Bosnia and Herzegovina during May – July 2007 [15].

Respondent Driven Sampling (RDS) was used to obtain a sample of hidden populations and to reach populations such as IDUs [16]. It is a form of chain-referral sampling. These chains consist of waves that penetrate deeper into the population in order to reach its hidden parts. RDS begins with a set of participants (“seeds”) who recruit their peers, people whom they know and who know them. The method is coupon-based recruitment process, accounts for homophily and non-random selection of initial respondents. In all three cities, six initial respondents (“seeds”), three female and three male, were selected in cooperation with NGOs. Respondents presented two sub-sample, one of 18–24 years and sub-sample of older than 25. Those “seeds” were the first “wave” of participants, who recruited the second wave. The number of waves was 7, in order to reduce selection bias (possibly caused of some “seeds” who were contacted by NGOs’ beneficiaries) and to reach deeper into the IDUs population and provide generalizability of the results [17]. The RDS method involves financial compensation, primarily for personal participation in the survey, but also for the recruitment of new participants. Respondents were allowed to recruit a maximum of three new participants. The primary incentive was 10 EURO (E), while the secondary incentive was 5E per recruited IDU.

Before the inclusion of respondents in survey, screener who was responsible for checking the legitimacy of participation explained the details of the research, aim of the research, procedures and the content of the questions. Anonymity, confidentiality and privacy of data were explained and guaranteed. Participants gave their written informed consent. A total of 750 IDUs participated in the survey and fulfilled the criteria. Participants had to be older than 18 years, injected drugs in the previous month, and living, studying or working in the cities where the survey was conducted for at least three months. The sample of 519 IDUs answered questions related to their oral health. All participants used heroin. There were 483 (93.1%) males and 36 (6.9%) females, 213 (41.0%) were from Sarajevo, 257 (49.5%) were from Zenica and 49 (9.4%) were from Banja Luka. The survey was approved by the Ethical Committees of the Clinical Centre of the University of Sarajevo, Cantonal Hospital in Zenica and Agency for Drugs of the Republic of Srpska (Banja Luka).

### Procedure

The data were obtained through face-to-face interviews using a structured questionnaire (Additional file 1: Questionnaire). There were 16 sections containing questions related to socio-demographic characteristics and HIV risk–taking behaviour among IDUs. The last section contained questions related to oral health: the number of missing teeth, problems with xerostomia, symptoms of common oral health problems, frequency of tooth brushing, frequency of visits to the dentist, the use of private or public dental services, the assessment of accessibility of dental services and self-report oral health status. A pre-test questionnaire was conducted in Banja Luka on a group of 10 IDUs, in order to determine appropriate form of the questions, their order and length of the interview. The interview took approximately 40 minutes plus time for a short introduction and counselling about blood test procedure, before moving to the next biological stage of the research.

The NGO facilities were used to interview the IDUs which were open each weekday for 6 hours and Saturdays for 4 hours. Working hours were adjusted to suit the participants and interviews were mainly conducted in the afternoon. At each research facility, trained teams were available, whose members were responsible for a particular phase of the research (e.g. practical skills and knowledge on interviewing techniques and confidentiality). NGOs which took part in this survey, mostly dealt with education, networking, exchange of information, publishing, work on improving the position of marginalized groups, specially in field of drugs, and protection of human rights and freedoms. One of NGOs provided prevention activities and psychotherapeutic services for drug users and their families.

### Variables

A total of 10 variables were analyzed as predictors of oral health. These variables were: age, gender, level of education, marital status, employment status, housing, duration of drug use, frequency of drug injection in the last month, frequency of tooth brushing and regular dental checkups. Housing was classified into two categories, where the code “1” represented better housing (living in own or parents’ apartment/house), and code “0” (rented house, friends’/relatives’ house, collective residence, street/park/abandoned building, institutions for the treatment of addiction, prisons and others). Oral health of the participants was coded as: bad or good oral health. Oral health variable was composed of questions about mouth and teeth condition; missing teeth; difficulty with chewing and swallowing food due to problems with teeth and dry mouth; and frequent feeling of dryness in mouth. Score values of the answers at abovementioned first three questions were recorded on the scale from 0 (the worst value) to 4 (the best value). The last question respondent could answer with “yes” or “no” (“no” included no, I do not know and missed answer). The total score was the sum of the individual scores and ranged from 0 to 13. Applying the cut-off value (median was 7) on the total score the participants were divided into two groups, with good (values 8 and more) or bad oral health (7 and less). A variable regular checkup at the dentist was obtained by dichotomization of answers to question “In the past year, how many times have you visited a dentist?” Participants who had answered that they visited dentist one or more times during past year, were coded as participants who regularly visited a dentist. A variable teeth brushing was obtained by dichotomization of answers to question “How often do you brush your teeth?”, while regular teeth brushing implied twice a day or after every meal.

### Statistical analysis

Data were expressed as frequencies (percentages) for categorical variables and as means ± SD or medians and range for continuous variables, respectively. Univariate analyses were conducted to assess the association between each independent variable and the outcome variable, bad or good oral health. All variables which were significantly associated with the outcome measure (p < 0.05) were entered into a multiple logistic regression model. The IBM SPSS Statistics 19 package was used for these analyses.

## Results

In total, 519 heroin IDUs with an average age of 28.80 ± 6.96 years were interviewed. The age of the participants ranged from 17 to 53 years. Almost two thirds of participants were single (303, 58.4%). Most had secondary education (348, 67.1%) or primary education (134, 25.8%), while 12 (2.3%) had college or faculty education. Twenty-five (4.8%) participants had no education. Most of the participants were unemployed (453, 87.3%). Eighty percent of participants lived in their own apartment/house or parents’ house. The average duration of drug use was 5.69 ± 5.06 years (range, 0–33 years), and the average frequency of drug injection in the last month was 5.43 ± 2.09 (range, 1–9). Only 39 (7.5%) participants had regular dental checkups. More than half of the participants (273, 52.6%) visited the dentist on a yearly basis, 101 (19.5%) once a year, 61 (11.8%) twice a year, and 113 (21.8%) three or more times a year. Participants preferred to visit the private dental sector than the public dental sector (39.3% vs. 24.9%, respectively). Both sectors were used by 79 (15.2%) of participants.

Socio-demographic characteristics and oral hygiene habits according to oral health are presented in Table 1. As shown in Table 1, after applying the univariate analysis, 7 out of 10 variables described the oral health of the participants. Those with bad oral health were significantly older, had a lower level of education, were more often unemployed, had a longer duration of drug use, frequently injected drugs during the last month, did not regularly brush their teeth, and did not go for regular dental checkups.

**Table 1:** Socio-demographic characteristics of the study participants

**Socio-demographic characteristics and oral hygiene habits**	**Bad oral health**	**Good oral health**	**p**
	**(n = 270)**	**(n = 249)**	
Age (years), mean ± SD	30.6 ± 7.5	26.8 ± 5.7	<0.001*
Gender, n (%)			0.345
Males	254 (94.1)	229 (92.0)	
Females	16 (5.9)	20 (8.0)	
Level of education, n (%)			<0.001*
No education	16 (5.9)	9 (3.6)	
Primary	96 (35.6)	38 (15.3)	
Secondary	152 (56.3)	196 (78.7)	
College/Faculty	6 (2.2)	6 (2.4)	
Marital status, n (%)			0.256
Married	42 (15.6)	35 (14.1)	
Permanent relationship	64 (23.7)	75 (30.1)	
Single	164 (60.7)	139 (55.8)	
Employment status, n (%)			0.001*
Full – time job	7 (2.6)	21 (8.4)	
Part – time job	13 (4.8)	17 (6.8)	
Unemployed	249 (92.2)	204 (81.9)	
Student	1 (0.4)	7 (2.8)	
Housing, n (%)			0.130
House/apartment (own or parents’)	209 (71.4)	206 (82.7)	
Other	61 (22.6)	43 (17.3)	
Duration of drug injection (years), median (range)	6 (0 – 33)	3 (0 – 23)	<0.001*
Frequency of drug injection in last month, mean ± SD	5.7 ± 2.1	5.2 ± 2.1	0.009*
Frequency of teeth brushing, n (%)			<0.001*
Do not brush teeth	59 (21.9)	6 (2.4)	
Brush teeth but not every day	70 (25.9)	32 (12.9)	
Once a day	73 (27.0)	88 (35.3)	
Twice a day	52 (19.3)	98 (39.4)	
After every meal	16 (5.9)	25 (10.0)	
Regular checkups at the dentist, n (%)			<0.001*
Do not go	262 (97.0)	218 (87.6)	
Go	8 (3.0)	31 (12.4)	

*Statistical significance was considered p < 0.05.

Multiple logistic regression analyses (Table 2) indicated that a higher level of education (OR = 0.56, 95% CI: 0.39-0.79), more frequent tooth brushing (OR = 0.59, 95% CI: 0.49-0.71) and regular dental checkups (OR = 3.30, 95% CI: 1.42-7.67) were predictors of good oral health. Older participants (OR = 1.06, 95% CI: 1.02-1.10), part–time employment (OR = 3.57, 95% CI: 1.02-12.20) and unemployment (OR = 3.23, 95% CI: 1.23-8.33, according to full-time employment as the referent category), and longer duration of drug use (OR = 1.06, 95% CI: 1.003-1.12) were predictors of bad oral health.

**Table 2:** Multiple logistic regression model with oral health as the dependent variable

**Independent variables**	**B**	**p**	**OR (95% CI)**
Age	0.057	0.003*	1.06 (1.02 – 1.10)
Level of education	-0.588	0.001*	0.56 (0.39 – 0.79)
Employment status			
Full – time job	(Ref.)		
Part – time job	1.262	0.046*	3.57 (1.02 – 12.20)
Unemployed	1.112	0.017*	3.23 (1.23 – 8.33)
Student	0.328	0.705	1.59 (0.14 – 16.67)
Duration of drug injection	0.057	0.037*	1.06 (1.003 – 1.12)
Frequency of drug injection in last month	0.055	0.260	1.06 (0.96 – 1.16)
Frequency of teeth brushing	-0.528	<0.001*	0.59 (0.49 – 0.71)
Regular checkups at the dentist	1.194	0.006*	3.30 (1.42 – 7.67)

B, regression coefficient, OR, adjusted odds ratio, 95% CI, 95% confidence interval.

*Statistical significance was considered p < 0.05.

## Discussion

In a sample of 519 heroin IDUs in Sarajevo, Banja Luka and Zenica in Bosnia and Herzegovina, we examined the relations between socioeconomic factors, drug use habits, and oral hygiene habits in participants with good and bad oral health.

Our results showed that older participants and those with a longer duration of drug use had a higher risk of developing bad oral health which is in accordance with previous research [5,18]. In addition, participants with part-time employment, unemployed and those who did not go for regular dental checkups had a 3-fold higher risk of developing bad oral health than participants with full–time jobs and who attended regular dental checkups. Researchers have shown that poor dental health among IDUs was related to untreated oral health problems. IDUs did not seek formal treatment except in emergency situations, took extra drugs for toothache, or ignored oral problems until they were placed in rehabilitation facilities [3,6,19,20]. In Bosnia and Herzegovina, there are no specialized treatment centers for drug users. Community mental health centers across the country provide outpatient treatment and counselling for drug users who are not their only target group [21]. Dental health problems could be resolved in public or private institutions; however, IDUs did not utilize services enough from neither of these two institutions. The reason could be to keep secret of their addiction.

The research conducted on 58 drug use participants at the Native American Health Centre in San Francisco showed that, even they received a voucher for a dental examination, they did not make an appointment for it [7]. The use of dental care facilities may depend on factors such as intensity of drug use, public versus private care, treatment experience, low self-esteem and dental anxiety [3,22].The researcher’s assumption was that IDUs may need different type of dental care services. Dental care could be provided as integral part of treatment addiction in rehabilitations centers. Dental professionals in public or private health centers should have enough knowledge to recognize the symptoms of drug abuse and provide the best possible dental treatment. This could lead to access to dental services on an equitable basis and more open communication between dentist and patient. Also, to develop adequate strategies to prevent and manage dental diseases for this population group should be challenge for public health professionals [7,23].

In 2007, Bosnia and Herzegovina characterized by a very high unemployment rate of 47.24%, statistically demonstrated increased income, however, at the same time food prices increased compared with 2006 [21]. In addition, national data showed that oral health in the general population and children was unsatisfactory and preventive measures among children in the first grade of primary school have been carried out since 2005. More than half of the total dental morbidity are diseases of the adult population (55%). In 2007 in this age group occurred change of the first two top diseases. So after many years diseases of the dental pulp and periapical tissues (K04) was on the first place (35.0%), while dental caries (K02) was in second place (33.0%) [21]. According to the data oral health in Bosnia and Herzegovina was the worst in the Europe [24] Regardless of the poor socioeconomic conditions in Bosnia and Herzegovina, heroin abusers not only have bad general health, but they also have significantly poorer oral health and oral hygiene practices. The situation in Bosnia and Herzegovina, in addition to poor living conditions, is complicated by the availability of drugs and their street price. The street price of heroin per gram varied from city to city, and is five time more expensive in Sarajevo than in Banja Luka (3 EUR) [25].

In this survey, participants who brushed their teeth more often (once or twice a day) had a reduced risk of developing bad oral health. The reason for this could be better living conditions as well as better personal hygiene (live in own or parents’ house). Infrequent tooth brushing is a common behaviour among drug users. Research found that IDUs brush their teeth less than once a day and do not accept the responsibility and obligations of dental treatment [3,7]. In our research, participants with a higher level of education had a lower risk of developing bad oral health which could be related to better knowledge regarding health.

Although Bosnia and Herzegovina has an HIV/AIDS infection rate of 0.01% and has a low HIV prevalence [26], solving the problems related to drug abuse is an important public health task. The country did not face the problem of drug use until 2007. There were only few centers for drug addiction treatment, including those which provide substitute therapy. There was lack or no continuous prevention programme at schools and in the community. The adopted state drug strategy was not implemented. Also, information system on drugs was still under development. The research on the biological and behavioural survey among injection drug users in Sarajevo, Banja Luka and Zenica provided evidence for further policy decisions and services development for IDUs. The poor dental health in this target group indicated the need for more collaboration within the community, e.g. between the social and dental health care sectors which should develop strategies to prevent and manage dental disease in this population. Another survey on IDUs using the RDS method was carried out in 2009, and progress was made when the ‘Protocol on Cooperation for the implementation of the Law on the Prevention and Combat of the Abuse of Narcotics - The evidence of drug addicts and abusers’ was signed in February 2010 [15]. With this protocol, state, NGO, and private sectors have an obligation to collect report and share information as well as to provide detoxification, drug treatment, rehabilitation, religion-based rehabilitation and other forms of drug treatment [11].

The results of our study must be viewed in the light of several limitations. First, RDS data questions were read to participants and some answers may have been biased, e.g. drug use. Data on oral health was based on participants’ self–reports and further work should be carried out in collaboration with dentists for an objective assessment of oral health. Although it is helpful to run weighted and unweighted analyses, we did not collect the RDS data that were needed to generate weights. Future investigations should include variables which more precisely describe oral health and problems (e.g. filled tooth index, decayed and missing teeth). Information on dental problems and oral health status were self-perceived as well as those on dental service use and may be subject to recall bias. Sample size was generally small and women represented only a small number of the respondents.

## Conclusions

These findings suggest that older participants, part–time employment, unemployment, longer duration of heroin injection and participants who did not have good oral hygiene habits such as frequent tooth brushing and regular dental checkups, had bad oral health. Planning services for IDUs should be based on collaboration between public health professionals who work on HIV prevention among IDUs and dentists which may lead to better programmes and oral health care for this disadvantaged group. Studies on oral health could also involve monitoring the behavior and health of other hidden populations such as commercial sex workers and the homeless. This would help to understand their needs and to direct the development of an adequate social response.

## Competing interests

The authors declare that they have no competing interests.

## Authors’ contributions

ZTS completed the interpretation of data, and carried out a critical revision of the manuscript for important intellectual content. RP did study concept and design and completed the interpretation of data. MSM participated in data integration and data analysis accuracy. GT and ZB were responsible for statistical analysis and data presented. All authors had full access to all data, read the manuscript and approved the final version.

## Pre-publication history

The pre-publication history for this paper can be accessed here:

http://www.biomedcentral.com/1471-2458/13/1202/prepub

## Supplementary Material

Additional file 1 Questionnaire. Click here for file
