# Supplementary material for: The oral health of heroin drug users: case study in Bosnia and Herzegovina
Source: BMC Public Health. 2013 Dec 19;13:1202. doi: 10.1186/1471-2458-13-1202 (PMC3878313; doi:10.1186/1471-2458-13-1202)
Supplement: Additional file 1 — Questionnaire. [file 1471-2458-13-1202-S1.pdf]

UNICEF, “Andrija Štampar School of Public Health”,
NGO Viktorija, NGO Margina, NGO UG PROI

Survey on prevalence of HIV, hepatitis B, hepatitis C, syphilis and risk behaviour among injecting drug users in Bosnia and Herzegovina

RDS LABEL

A. DEMOGRAPHIC CHARACTERISTICS

First, I would like to ask you some questions about you and your surroundings.

01. How old are you?

[ ][ ] years old

02. What is your gender?
(do not read the answers)

1. Male
2. Female

03. What is your highest level of education?

1. No education
2. Elementary
3. High school
4. College

04. Are you currently...?

1. Married
2. In a steady relationship
3. Single

05. Where did you live during the past three months?
(do not read the answers)

! CHOOSE ONLY ONE ANSWER

1. In your own house or apartment
(house or apartment of your spouse or partner)
2. In a rented house or apartment
(house or apartment of your spouse or partner)
3. In your parents' house or apartment
4. In someone else's house or apartment
(of your relatives, friends)
5. Dormitory
(students' dormitory, etc.)
6. At no permanent location
(e.g. street, park, deserted building)
7. In an institution for treatment of drug addiction

8. Prison
9. Another location......................................
10. No answer

06. Are you currently employed?

1. I have a full-time job
2. I have a part-time job
3. I am unemployed
4. I am a student

B. DRUG USE

The following questions are related to the type of drugs you are using and frequency of use.

07. How old were you when you first used drugs of any kind?

| | | years old

08. Which drug did you use then?
…………………………………….. (drug name)

09. How old were you when you first injected drugs?

| | | years old

10. How long have you been injecting drugs?

| | | years

| | | months (if less than a year)

Remark:
Less than a month = 00, Don't know = 88, No answer = 99

11. How often did you inject drugs during the past month?

1. Once
2. 2-3 times
3. Once a week
4. 2-3 times a week
5. 4-6 times a week
6. Once a day
7. 2-3 times a day
8. 4 or more times a day
9. No answer

12. On the last day you injected drugs, how many times did you do it?

| | | times

Remark:
Don't know = 88, No answer = 99

13. On which locations did you inject drugs during the past month?
(show card A)

! IT IS POSSIBLE TO CHOOSE SEVERAL ANSWERS

1. In a private house or apartment
2. In a public place, e.g. café or shop
3. In a public toilet
4. In a dealer's house or apartment
5. On a street or in a park
6. In a deserted building
7. In a basement of a building
8. In a lobby of a building
9. In a shooting gallery or in another place for gathering of drug addicts
10. In prison
11. Other...
12. No answer

14. Remember when you were younger than 18, at which locations did you inject drugs most often then?
(show card A)

! IT IS POSSIBLE TO CHOOSE SEVERAL ANSWERS

13. In a private house or apartment
14. In a public place, e.g. café or shop
15. In a public toilet

16. In a dealer's house or apartment
17. On a street or in a park
18. In a deserted building
19. In a basement of a building
20. In a lobby of a building
21. In a shooting gallery or in another place for gathering of drug addicts
22. In prison
23. Other...
24. No answer

I will now ask you some questions about the drugs that you have used so far. For each drug that I mention I will first ask you whether you injected it during the past months and then I will ask you which drug you injected most frequently during the past month.

Firstly, concerning the drugs mentioned below, please state: (show card B)

15. Which drugs did you inject during the past month?
(allow multiple answers)

16. Which drug did you inject most frequently during the past month?
(allow only one answer)

DRUG		15.	16.
1	Heroin		
2	Cocaine		
3	Heroin and cocaine together		
4	Amphetamine		
5	Morphine		
6	Opium		
7	Methadone<br>ampoule for injection		
8	Methadone – liquid		
9	Trodon (Tramadol) - liquid		
10	Ecstasy		
OTHER (please specify)			
11			
12			
13			
14			

C. INJECTING PRACTICES AND SHARING OF INJECTING EQUIPMENT

17. During the past month, from how many different persons did you take previously used needles and/or syringes you then used for your own injecting?

[ ][ ] persons

Remark:
Don't know = 88, No answer = 99

During the past month, did you:

18. Inject drugs using a syringe after someone else filled it with drugs from his syringe?
(filling in from below/filling in from above/sharing)

1. Yes
2. No
3. Don't know
4. No answer

19. used a filter or cotton wool through which someone else inserted drugs using his needle/syringe?

1. Yes
2. No
3. Don't know
4. No answer

20. suck your drug solution into a syringe from a mixing/cooking vessel (spoon or glass

container) previously someone else sucked in from?

A. Yes
B. No
C. Don't know
D. No answer

21. used the water previously used by someone else for rinsing or cleaning of syringes?

A. Yes
B. No
C. Don't know
D. No answer

22. How many different persons used your used needle and/or syringe during the past month?

[ ][ ] persons

Remember the last time you injected using a needle and/or syringe previously used by someone else

23. How long ago did this happen?
(do not read the answers)

[ ][ ] days
[ ][ ] weeks
[ ][ ] months
[ ][ ] years

[ ] never

!	If the answer is „never", skip the following questions and go to Section D.

24. Who was the person whose needle and/or syringe you used the last time you injected with a needle and/or syringe previously used by someone else?
(mark only one answer)

1. An unknown person, someone you just met
2. A casual acquaintance
3. A close friend
4. A sexual partner
5. A family member or a relative
6. A dealer
7. Other (please specify)...................

25. Did you try to clean in any way the needle/syringe you borrowed?

1. Yes
2. No
3. Don't know
4. No answer

!	If the answer was 2, 3 or 4, skip the following questions and go to Section D.

26. In which way did you try to clean the needle/syringe?
(do not read the answers)

1. With cold water
2. With warm water
3. With hot water
4. With boiling water from the pot
5. With soap or detergent
6. With bleach
7. With alcohol
8. Other (please specify) ……...........................

D. USING NEW NEEDLES AND SYRINGES

Now I would like to ask you something about your use and provision of new needles/syringes

27. What is the average number of times you use a needle/syringe for injecting before you throw it away?

[ ] times

Remark:
Only once = 001, Don't know = 888,
No answer = 999

> ! If the answer was „only once", skip the following questions and go to Question 30.

28. How often do you clean an already used needle/syringe?

1. Always (100%)
2. Most of the times (75%)
3. Half of the times (50%)
4. Sometimes (25%)
5. Rarely (below 10%)
6. Never

26. How do you usually clean an already used needle/syringe?
(do not read answers)

1. With cold water
2. With warm water
3. With hot water
4. With boiling water from the pot
5. With soap or detergent
6. With bleach
7. With alcohol
8. Other (please specify)..........................

I will now ask you some questions related to provision of syringes (show card C)

30. On which of the following places did you get needles/syringes during the past month?
(allow multiple answers)

31. What is your main source for provision of syringes?
(allow only one answer)

PROVISION OF SYRINGES		30.	31.
1	Buying in a pharmacy		
2	Stealing in a pharmacy, hospital or shop		
3	In NGO ________		
4	On the places where I inject		
5	On the street		
6	From friends who are not injecting drug users		
7	From friends who are injecting drug users		
8	From family		
In a health institution (please specify)			
9			
OTHER (please specify)			
10			
11			

32. How many new needles/syringes did you get during the past week?

[ ] number

Remark:
Don't know = 88, No answer =999

33. How many of those (from the previous question) did you keep for your own use?

[ ] number

Remark:
Don't know = 88, No answer =999

34. How many of them did you give or sell to others?

[ ] number

Remark:
Don't know = 88, No answer =99

E. POLICE AND/OR PRISON EXPERIENCE

Now I would like to ask you something about your experiences with the police.

35. How many times did the police stop you and ask for your ID during the past year?

[ ] times

Remark:
Don't know = 888, No answer = 999, Never = 000

36. How many times were you arrested or brought into a police station during the past year?

[ ] times

Remark:
No answer = 999, Never = 000

> **!** If the answer is „never“, skip the following question and go to Question 38.

37. During the past year, were you brought into a police station for one of the following reasons: (show card D)

Remark:
Allow multiple answers

1	Possession of drugs	yes	no
2	Use of drugs	yes	no
3	Carrying of sterile needles or syringes	yes	no
4	Carrying of used needles or syringes	yes	no
5	Offering of sexual services in return for money, drugs or goods	yes	no
6	Theft/Robbery	yes	no
7	Selling or buying drugs	yes	no
OTHER (please specify)			
8			

Now I would like to ask you something about your experiences with prison.

38. Have you ever been to prison?

1. Yes
2. No
3. No answer

> **!** If the answer was 2 or 3, skip the following questions and go to Section F.

39. How many times you were in prison?

[ ] times

Remark: Don't know = 88, No answer = 99

40. State the month and the year of your last coming out of prison?

	month
	year

Remark: Don't know = 88, No answer = 99

41. Did you inject drugs during your last stay in prison?

1. Yes
2. No
3. No answer

F. SEXUAL PRACTICES

I will now ask you some questions about your sexual practices.

42. Have you ever had a sexual intercourse? (vaginal, anal and/or oral sex))

1. Yes
2. No
3. No answer

> ! If the answer was 2 or 3, skip the following question and go to Section L.

43. How old were you at the time of your first sexual intercourse (vaginal and/or anal sex)?

[ ] years old

44. Did you have a sexual intercourse during the past year?

1. Yes
2. No
3. No answer

> ! If the answer was 2 or 3, skip the following questions and go to Section K.

45. With how many different persons did you have a sexual intercourse (vaginal and/or anal sex) during the past year?

[ ] persons

Don't know = 888, No answer = 999, None = 000

G. SEXUAL INTERCOURSE WITH REGULAR PARTNERS

46. Did you have a sexual intercourse with your regular partner (spouse, boyfriend/girlfriend) during the past year?

1. Yes
2. No

> ! If the answer was 2, skip the following questions and go to Section H.

47. How often did you use condoms with your regular partner during the past month?

1. Always (100%)
2. Most of the time (75%)
3. Half of the time (50%)
4. Sometimes (25%)
5. Rarely (below 10%)
6. Never
7. I have not had a sexual intercourse with my regular partner during the past month

! If the answer was 7, skip the following questions and go to Section H.

48. During your last sexual intercourse (vaginal and/or anal) with your regular partner, did you use a condom?

1. Yes
2. No
3. No answer

49. Is your regular partner an injecting drug user?

1. Yes
2. No
3. Don't know
4. No answer

H. SEXUAL INTERCOURSE WITH CASUAL PARTNERS

50. Did you have a sexual intercourse with a casual partner (person with whom you are not in a relation) during the past year?

1. Yes
2. No

51. With how many casual partners did you have a sexual intercourse during the past month?

[ ] partners

! If the answer was „none“, skip the following questions and go to Section I.

52. Were there any injecting drug users among those casual partners?

1. Yes
2. No
3. Don't know

53. How often did you use condoms with your casual partners during the past month?

1. Always (100%)
2. Most of the time (75%)
3. Half of the time (50%)
4. Sometimes (25%)
5. Rarely (below 10%)
6. Never

54. Did you use a condom when you had your last sexual intercourse with a casual partner?

1. Yes
2. No
3. No answer

55. Is that casual partner an injecting drug user?

1. Yes
2. No
3. Don't know
4. No answer

I. SEXUAL INTERCOURSE WITH SOMEONE YOU PAID

56. During the past year, have you had a sexual intercourse (vaginal and/or anal) with someone you paid for sex?

1. Yes
2. No

! If the answer is 2, skip the following questions and go to Section J.

57. During the past month, with how many persons you paid for sex did you have a sexual intercourse (vaginal, anal) or oral sex?

[ ] persons

> ! If the answer was „none“, skip the following questions and go to Section J.

58. During the past month, how often did you use condoms with persons you paid for sex?

    1. Always (100%)
    2. Most of the time (75%)
    3. Half of the time (50%)
    4. Sometimes (25%)
    5. Rarely (below 10%)
    6. Never

59. Did you use a condom when you had your last sexual intercourse (vaginal and/or anal) with a person you paid for sex?

    1. Yes
    2. No
    3. Don't know
    4. No answer

60. Is that person you last had paid sex with an injecting drug user?

    1. Yes
    2. No
    3. Don't know
    4. No answer

J. SEX WORK

61. Have you ever taken money, goods or drugs in exchange for sexual intercourse (vaginal and/or anal) or oral sex?

    1. Yes
    2. No

> ! If the answer was 2, skip the following questions and go to Section K.

62. How old were you when you first took money, goods or drugs in exchange for sexual intercourse (vaginal and/or anal) or oral sex?

    Remark:
    Don't know = 88, No answer = 99, None = 00

    [ ] years old

63. During the past year, did you have a sexual intercourse with someone who paid you for sex?

    1. Yes
    2. No

64. With how many persons did you have paid sex during the past month:

    [ ][ ][ ]

65. Did you use a condom when you had your last sexual intercourse (vaginal and/or oral) with a client?

    1. Yes
    2. No
    3. No answer

66. What is your most common way to get clients? (do not read the answers, only one answer possible)

    1. Through newspaper ads
    2. In a bar
    3. In a brothel
    4. Through internet
    5. Other (please specify).............

Now try to remember your last sexual intercourse.

67. With whom did you have this last sexual intercourse (vaginal and/or anal)?

1. With your regular partner (spouse, boyfriend/girlfriend)
2. With a casual partner
3. With someone you paid for sex
4. With someone who paid you for sex

68. During the past year, have you had a sexual intercourse with a person of the same sex?

1. Yes
2. No

K. CONDOMS AND REPRODUCTIVE HEALTH

69. Where can one buy or get condoms around here?
(do not read answers)

Remark:
Allow multiple answers

1. Can't think of any place
2. Pharmacy
3. Shop/Drugstore
4. Tobacco Shops/Gas Stations
5. NGO (please specify)...................................
6. Other...

70. What is your main source of supply with condoms?
(do not read the answers)

Remark:
Choose only one answer

1. I don't use condoms
2. Can't think of any place
3. Pharmacy
4. Store/Drugstore
5. Tobacco Shops/Gas Stations
6. Outreach workers
7. Other.......................

I will now ask you some questions about sexually transmitted diseases.

71. Have you been diagnosed with a sexually transmitted disease during the past year?

1. Yes
2. No
3. No answer

72. Have you had wounds, swellings or unusual genital discharge during the past year?

1. Yes
2. No
3. No answer

73. What did you do the last time you had wounds, swellings or unusual genital discharge?
(do not read answers)

1. Didn't do anything
2. I went to a state health care institution for checkup and treatment
3. I went to a private health care institution for checkup and treatment

4. I went to a pharmacy to buy medications (without consultation with a doctor)
5. I treated it at home (without medications)
6. I stopped having sexual intercourses
7. I turned to an outreach worker for advice
8. Other..

L. HIV TESTING

74. Do you know where you can be tested for HIV?

    1. Yes
    2. No
    3. No answer

75. Have you ever been tested for HIV?

    1. Yes, once
    2. Yes, multiple times
    3. No
    4. No answer

> ! If the answer was 3 or 4, skip the following questions and go to Question 80.

76. When was the last time you were tested for HIV?

Write down the year:

Remark:
Don't know = 888, No answer = 999

77. Was that a voluntary HIV testing or you were required to do it?

    1. Voluntary testing
    2. Required testing

78. Where you were last tested for HIV?

Remark:
Mark only one answer

1. Clinic/Hospital for Infectious Diseases
2. NGO/counseling service
3. Public Health Institute
4. Prenatal Clinic
5. Private Clinic/Laboratory
6. Prison
7. Other...

79. Do you know the result of your last HIV test (do not tell me what it was!)?

    1. Yes
    2. No
    3. No answer

80. Have you ever been tested for hepatitis C?

    1. Yes
    2. No
    3. No answer

> ! If the answer was 2 ili 3, skip the following questions and go to Section M.

81. When was the last time you were tested for hepatitis C?

Write down the year:

Remark:
Don't know = 888, No answer = 999

82. Do you know the result of your last test for hepatitis C?

    1. Yes
    2. No
    3. No answer

M. KNOWLEDGE OF HIV

I will now ask you some questions about the way of transmission of the HIV virus.

83. Can HIV be avoided by having a sexual intercourse with only one, faithful and uninfected sexual partner?

    1. Yes
    2. No
    3. Don't know

84. Can HIV be avoided by a proper use of condoms?

    1. Yes
    2. No
    3. Don't know

85. Can HIV be transmitted by using an already used needle and/or syringe?

    1. Yes
    2. No
    3. Don't know

86. Can a person who looks healthy have HIV?

    1. Yes
    2. No
    3. Don't know

87. Can HIV be transmitted by sharing food with a person infected by HIV?

    1. Yes
    2. No
    3. Don't know

88. When it comes to likelihood of HIV infection, how much do you think you are exposed to the risk of HIV infection?

    1. There is no risk
    2. The risk is small
    3. The risk is moderate
    4. The risk is big
    5. The risk is extremely big

N. DRUG ADDICTION TREATMENT

I will now ask you some questions about your experience with drug addiction treatment.

89. Do you know of any organisations involved in harm reduction for injecting drug users in your town?

    1. Yes
    2. No
    3. No answer

90. Have you been using services of those organisations during the past year?

    1. Yes
    2. No
    3. No answer

91. In the last year, have you received sterile needles from an NGO or some public institution (e.g. infectious disease clinic)?

    1. Yes
    2. No
    3. No answer

92. Have you ever undergone a treatment that would help you change, decrease or stop using drugs?

    1. Yes
    2. No
    3. No answer

! If the answer was 2 or 3, finish the interview and thank the interviewee for participation.

93. How old were you the first time you underwent treatment in order to change, decrease or stop using drugs?

[ ] years old

Remark:
No answer = 99

94. During the past year, where did you undergo treatment in order to change, decrease or stop using drugs?
(do not read the answers)

Remark:
Allow multiple answers

1. Clinic counselling/Psychotherapy
2. Hospital detoxication with use of medications
3. Hospital rehabilitation programme
4. Clinic rehabilitation programme
5. Selfhelp (I am trying to get off by myself)
6. Community support: church
7. Community support:: family
8. Community support: NGO/counselling services
9. Other (please specify)................................
10. I didn't undergo treatment during the past year

95. When was the last time you underwent treatment to change, decrease or stop using drugs?

[ ] year

Remark:
No answer = 99

96. Are you currently under treatment that helps you change, decrease or stop using drugs?

1. Yes
2. No
3. No answer

! If the answer was 2 or 3, finish the interview and thank the interviewee for participation.

97. What kind of treatment are you currently under?
(do not read the answers)

Remark:
Allow multiple answers

1. Clinic counselling/Psychotherapy
2. Hospital detoxication with use of medications
3. Hospital rehabilitation programme
4. Clinic rehabilitation programme
5. Selfhelp (I am trying to get off by myself)
6. Community support: church
7. Community support: family
8. Community support: NGO/counselling services
9. Other (please specify)....................................
10. I didn't undergo treatment during the past year

NETWORK SIZE OF PARTICIPANTS

FILLED OUT BY COUNSELLOR/INTERVIEWER

We would like to ask you a few questions about your the people you know:

1. How many persons who inject drugs do you know, who live, work or study in the area of Banja Luka, and who have injected drugs during the last month? By knowing, we refer to persons whose name is familiar to you, as well as yours to them, and whom you have seen in the last 3 months.

   ☐

2. How many persons of those listed in the previous questions are younger than 16?

   ☐

3. The person who gave you the recruitment coupon is your: (do not read the answers)
   1. Friend
   2. Acquaintance
   3. Relative
   4. The first time I saw that person was when she/he gave me the coupon

4. Why did you personally decided to take the coupon and participate in this survey? (do not read the answers)
   1. Because of the financial incentive
   2. My acquaintance asked me to
   3. I want to get an advice about HIV/AIDS
   4. I want to get an HIV test
   5. I want to get tested for STIs
   6. Other (describe): _____________

dsUNICEF, NVO Viktorija, NVO Margina, UG PROI

Oral health and oral hygiene habits among injection drug users in Bosnia and Herzegovina

RDS LABEL

1. How often do you brush your teeth:
   1. Not every day
   2. Once a day
   3. Twice a day
   4. After every meal
   5. I do not brush my teeth
   6. I do not know

2. How would you describe the health condition of your mouth and teeth:
   1. Very good
   2. Good
   3. Neither good nor bad
   4. Bad
   5. Very bad
   6. I do not knoe

3. How many missing teeth do you have:
   1. I do not have any of my teeth
   2. More than 10 teeth
   3. 6 – 10 teeth
   4. 1 – 5 teeth
   5. I have all my teeth
   6. I do not know

4. In the past year, how many times have you visited a dentist?
   1. Neither one
   2. Once
   3. Twice
   4. Three or more times
   5. I do not remember

. 5. How long has it been since you last visited a dentist?

   1. <1 year
   2. ≥1<2 years
   3. ≥2<5 years
   4. ≥5 years
   5. I do not remember

6. What is the main reson of your last visit to a dentist:
   1. Check up
   2. Something was wrong (concerns or pain)
   3. Continuation of previous treatment
   4. Something else ....

7. Do you often feel that your mouth is dry?
   1. Yes Da
   2. No Ne
   3. I do not know

8. Due to problems with teeth and dry mouth (xerostomia, changes in the mucosa, ulcers, bleeding), how often do you have problems with chewing and swallowing food or are you limited in the amount of food you can eat?
   1. Always
   2. Often
   3. Sometimes
   4. Rarely r
   5. Newer

9. Do you visit the dentist:
   1. I do not visit the dentist
   2. I visit private dental services
   3. I visit public dental servise
   4. I visit both these categories of *services*

10. Have you experienced the dentist refused to provide treatment:
    1. Yes
    2. No

Thank you very much for your time and participation in survey
